# Quantification of structural changes in the corpus callosumin children with profound hypoxic–ischaemic brain injury

**DOI:** 10.1007/s00247-015-3444-3

**Published:** 2015-09-24

**Authors:** Stavros M. Stivaros, Mark R. Radon, Reneta Mileva, Daniel J. A. Connolly, Patricia E. Cowell, Nigel Hoggard, Neville B. Wright, Vivian Tang, Ann Gledson, Ruth Batty, John A. Keane, Paul D. Griffiths

**Affiliations:** Academic Unit of Paediatric Radiology, Royal Manchester Children’s Hospital, Central Manchester University Hospitals NHS Foundation Trust, Manchester Academic Health Science Centre, Manchester, UK; Centre for Imaging Sciences, Institute of Population Health, University of Manchester, Manchester, UK; Department of Neuroradiology, The Walton Centre NHS Foundation Trust, Liverpool, UK; School of Computer Science, University of Manchester, Manchester, UK; Department of Neuroradiology, Sheffield Children’s Hospital NHS Foundation Trust, Sheffield, UK; Department of Human Communication Sciences, University of Sheffield, Sheffield, UK; Academic Unit of Radiology, University of Sheffield, C Floor, Royal Hallamshire Hospital, Glossop Road, Sheffield, S10 2JF UK

**Keywords:** Corpus callosum, Children, Hypoxic–ischaemic brain injury, Magnetic resonance imaging, Support vector machine analysis

## Abstract

**Background:**

Birth-related acute profound hypoxic–ischaemic brain injury has specific patterns of damage including the paracentral lobules.

**Objective:**

To test the hypothesis that there is anatomically coherent regional volume loss of the corpus callosum as a result of this hemispheric abnormality.

**Materials and methods:**

Study subjects included 13 children with proven acute profound hypoxic–ischaemic brain injury and 13 children with developmental delay but no brain abnormalities. A computerised system divided the corpus callosum into 100 segments, measuring each width. Principal component analysis grouped the widths into contiguous anatomical regions. We conducted analysis of variance of corpus callosum widths as well as support vector machine stratification into patient groups.

**Results:**

There was statistically significant narrowing of the mid–posterior body and genu of the corpus callosum in children with hypoxic–ischaemic brain injury. Support vector machine analysis yielded over 95% accuracy in patient group stratification using the corpus callosum centile widths.

**Conclusion:**

Focal volume loss is seen in the corpus callosum of children with hypoxic–ischaemic brain injury secondary to loss of commissural fibres arising in the paracentral lobules. Support vector machine stratification into the hypoxic–ischaemic brain injury group or the control group on the basis of corpus callosum width is highly accurate and points towards rapid clinical translation of this technique as a potential biomarker of hypoxic–ischaemic brain injury.

## Introduction

**Fig. 1:**
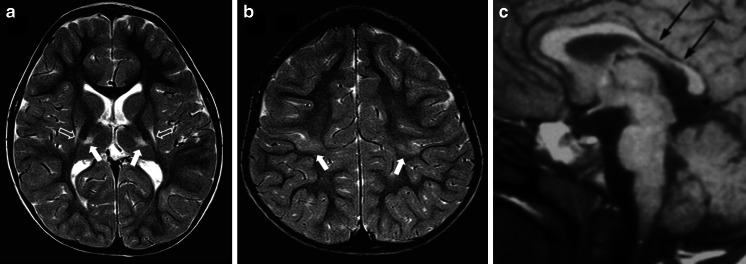
MR images in a 6-year old boy with dyskinetic cerebral palsy who has the typical neuroimaging findings of acute profound hypoxic–ischaemic brain injury. **a, b** Axial T2-weighted MR images at the level of the basal ganglia (**a**) and peri-rolandic cortex (**b**) show gliosis and reduction in volume of the putamina (*open arrows*), thalami and paracentral lobules (*solid arrows*). **c** A mid-sagittal T1-weighted MR image shows a focal reduction in thickness in the posterior part of the body of the corpus callosum (*arrows*)

Reduced blood supply or oxygen delivery around the time of birth is often termed “birth asphyxia” and is a cause of neonatal encephalopathy. In some cases there is lasting brain injury visible on MR imaging, usually in children who have sustained moderate or severe hypoxia/ischaemia. The pattern of such hypoxic–ischaemic brain injury on subsequent brain MR imaging studies depends on several factors, but most important are the gestational maturity and the severity and duration of the hypoxic–ischaemic event. Children with hypoxic–ischaemic brain injury who are born at or near term (37 weeks or more) usually have one of two patterns of injury. If the hypoxic–ischaemic event is short-lasting (10–25 min) but severe (e.g., following uterine rupture) the injury tends to involve the basal ganglia, thalami and paracentral lobules and is called acute profound hypoxic–ischaemic brain injury (Fig. 1) [1]. If the hypoxic–ischaemic event is more prolonged, with less severe reduction in blood supply or oxygen delivery (e.g., incomplete placental abruption) the parasagittal watershed regions of the cerebral hemispheres tend to be injured and this is called prolonged partial hypoxic–ischaemic brain injury [1]. Mixed patterns of acute profound and prolonged partial hypoxic–ischaemic brain injury can occur. Children affected by either of those injuries are likely to incur some form of cerebral palsy.

In this paper we describe a group of children with acute profound hypoxic–ischaemic brain injury on the basis of perinatal history, clinical outcome and MR imaging findings. Empirical observations suggest that there is specific corpus callosum volume loss in these children, although the changes in some cases can be subtle [1].

We quantitatively analysed the dimensions of the corpus callosum and hypothesised that the corpus callosum has a reduced size secondary to acute profound hypoxic–ischaemic brain injury and that the volume loss is focal, involving the region that carries the commissural axons from the paracentral lobules.

## Materials and methods

### Subjects

This retrospective study was conducted in a paediatric tertiary referral centre involving all cases that met the criteria during a 4-year period (*n* = 13). The study was conducted as part of an audit study registered with, and with the approval and ethical review of, Sheffield Children’s Hospital, Sheffield, England, under the auspices of investigating improvements in patient care. The patient group included children sequentially referred for clinical imaging; all of these children had cerebral palsy on clinical examination, birth histories and MR findings consistent with profound hypoxic–ischaemic brain injury acquired at or near term on the basis of features listed in the international consensus statement [2]. A summary of the cases is provided in Table 1. That group was compared with a pathology control group consisting of 13 age-matched children with idiopathic developmental delay who also had MR scans for clinical purposes. The children with developmental delay had no focal neurological symptoms or signs, no microcephaly, no history of epilepsy and normal MR examinations as assessed by experienced paediatric neuroradiologists (S.S. 6 years, D.J.A.C. 12 years, P.D.G. 20 years experience as tertiary level paediatric neuroradiologists).

**Table 1:** Clinical and radiologic summary of the children with acute profound hypoxic–ischaemic brain injury

Patient number	Gender	Weeks’ gestation^b^	Apgar at 5 min	Umbilical blood pH	Cerebral palsy type	Age at MRI (y)	Damage to putamen	Damage to thalamus	Damage to PCWM	Other sites of damage
1^a^	M	39	4	Not recorded	Dyskinetic	15	Yes	Yes	Yes	Vermis, Hippocampi
2	M	38	3	Not recorded	Dyskinetic	10	Yes	Yes	Yes	STN
3 ^a^	M	40	5	6.9	Spastic	15	Yes	Yes	Yes	Vermis
4	M	36	0	6.6	Spastic	3	Yes	Yes	Yes	Vermis, hippocampi
5 ^a^	F	41	3	Not recorded	Spastic	3	Yes	No	Yes	OR
6 ^a^	M	39	4	6.7	Dyskinetic	6	Yes	Yes	Yes	Vermis, hippocampi
7 ^a^	M	39	6	Not recorded	Spastic	2	Yes	Yes	Yes	OR, hippocampi
8 ^a^	M	39	4	6.9	Spastic	2	Yes	Yes	Yes	Vermis, STN
9 ^a^	M	39	3	Not recorded	Dyskinetic	2	Yes	Yes	Yes	OR, STN
10	M	40	2	6.8	Dyskinetic	5	Yes	Yes	Yes	Hippocampi, STN
11 ^a^	F	41	4	6.9	Spastic	3	Yes	Yes	Yes	Vermis, OR, STN
12 ^a^	M	40	5	Not recorded	Spastic	1	Yes	Yes	Yes	OR
13 ^a^	M	40	4	6.7	Dyskinetic	3	Yes	Yes	Yes	OR, STN

^a^ Indicates children also reported for subthalamic nucleus assessment [14]

^b^All children had seizure onset in the first day of life

*OR* optic radiations, *PCWM* paracentral white matter, *STN* subthalamic nucleus

### MR imaging

All children were imaged on the same 1.5-T superconducting magnet MR system (Infinion; Philips Medical Systems, Best, the Netherlands). The imaging protocol consisted of multi-planar, multi-sequence imaging but always included whole-brain imaging with a 3-D volumetric spoiled gradient echo T1-weighted sequence using a standard proprietary head coil (repetition time/echo time 15/4.4 ms; isotropic resolution of 1 mm; bandwidth 31.25 and pre-pulse inversion recovery-prepared at 450 ms), which was used for further analysis in this report. No parallel acquisition was used and there was a linear k space trajectory.

### Image analysis

A proprietary workstation was used to create a mid-sagittal section from the T1-W volume data, oriented such that the pituitary gland, infundibulum and cerebral aqueduct were present on the section. Image processing was performed using locally developed software in the Visual C# programming environment (Microsoft Corp., Redmond, WA). A region of interest was defined around the corpus callosum on the midline-sagittal image by manual planimetry, with best efforts to exclude the fornix from the region of interest. The software automatically divided the region of interest into 100 radial segments by placing 99 percentile slices along its axis of the corpus callosum using previously described methods [3–6] modified by the addition of an automated version of the optimisation process. The software measured perimeter, centre-line length (as given by the mid-points of the 99 slices), area, and centile thickness of the corpus callosum.

### Statistical analysis

The Shapiro–Wilk test for normality was used to assess the distributions of patient ages and the corpus callosum lengths and areas. The paired Student’s *t*-test was used to compare the normally distributed corpus callosum lengths and areas of the acute profound hypoxic–ischaemic brain injury and pathology control groups. Because of the large number of highly correlated variables (widths), principal components analysis followed by varimax rotation [7] was applied to group the widths into a smaller number of descriptive components (or factors). Components were retained for analysis if they explained more than 1% of the total variance. The varimax rotation was used to output factors that were heavily loaded on the minimum number of widths, with the intention of demonstrating clear regions. This method represents a simplified form of the factor analysis presented in earlier work using this method [3–6]. Factor scores were compared using an analysis of variance with statistical significance set at the 95% level. The null hypothesis was that any differences in factor widths between the hypoxic–ischaemic brain injury and the control group were simply caused by random variance in callosal width. Statistical analysis was performed using R software (The R Foundation for Statistical Computing, Vienna, Austria) and Microsoft Excel (Microsoft Corp., Redmond, WA). These data were mapped onto an anatomical outline of the corpus callosum by identifying each region according to the eigenvalue with the highest coefficient for that centile.

To assess the technique as the basis for possible clinical translation, we then conducted a support vector machine analysis using the centile callosal widths for each child as input, looking to stratify between hypoxic–ischaemic brain injury and normal control groups. Support vector machine analysis is a machine learning technique that looks for the optimal hyperplane for linearly separable patterns, in this case hypoxic–ischaemic brain injury or control. Support vector machines can be applied where the patterns are not linearly separated, in other words in cases where a cluster analysis might not yield optimal separation between groups.

All experiments were run using the WEKA machine learning package (University of Waikato, Hamilton, New Zealand). The well-known sequential minimal optimisation algorithm [8] was used to train the classifier. To address the possibility of overfitting given the relative smallness of the dataset, 10-fold cross validation was used. To measure classifier performance the standard approaches were used: accuracy and error rate, confusion matrix, sensitivity and specificity, precision and recall, F-score and receiver operator characteristic curve [9]. For the results described pertaining to hypoxic–ischaemic brain injury/control classification we used sequential minimal optimisation algorithm with a linear kernel: K(x,y) = <x,y>. Whilst other kernels were used, the linear kernel gave the best results.

## Results

We included 13 children in the hypoxic–ischaemic brain injury group (median age 3 years, range 1–15 years; 84.6% male), and compared them to 13 age-matched controls with developmental delay but normal brain imaging (median age 3 years, range 10 months–17 years; 84.6% male). These children had MR imaging between April 20015 and February 2009.

**Fig. 2:**
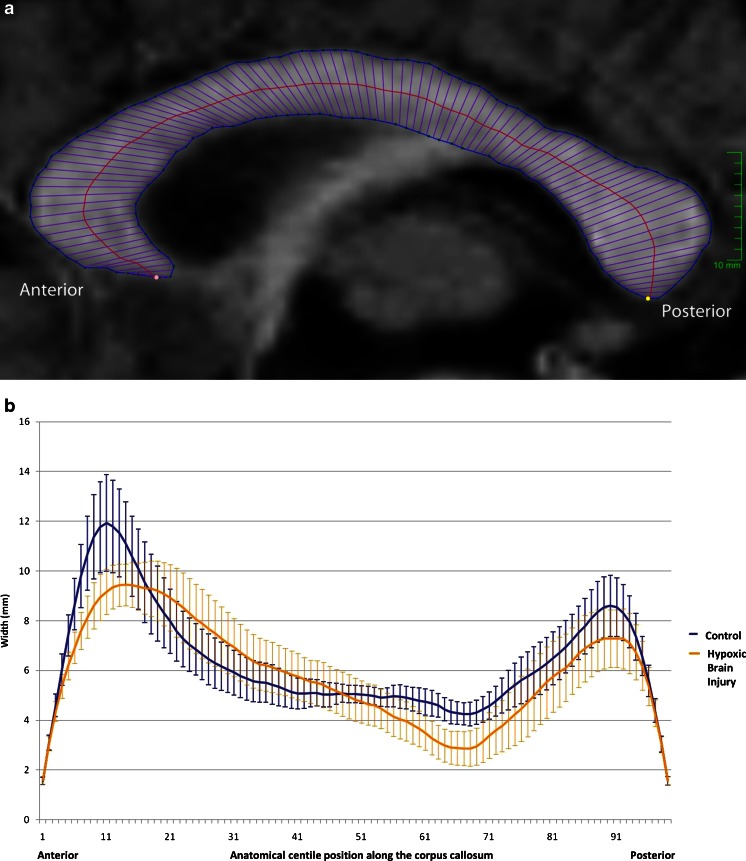
**a** A midline sagittal T1-weighted image showing a normal corpus callosum from a 6 year old age matched control child. The image also shows the placement of regions of interest and 99th percentile widths, with centreline based on the Denenberg technique [3]. **b** The width profiles (95% CI of the mean) for each centile generated for the control cases (*blue*) and the age-matched profound hypoxic–ischaemic brain injury cases (*yellow*)

The age-paired *t*-test for differences in gross corpus callosum geometrical features did not demonstrate a statistically significant difference between the control group and the acute profound hypoxic–ischaemic brain injury group for corpus callosum area (*P* = 0.37), perimeter (*P* = 0.40) or centreline length (*P* = 0.26). The corpus callosum width profiles for the two groups (Fig. 2) suggest reduced thickness of the corpus callosum in the acute profound hypoxic–ischaemic brain injury group in the anterior region (centiles 8–15) and in the mid-posterior body (centiles 61–70), with a maximum mean difference of 1.47 mm in centile 63. The absolute differences, however, were small with wide confidence intervals, and no individual centile width showed a statistically significant difference between the groups after Bonferoni correction.

**Fig. 3:**
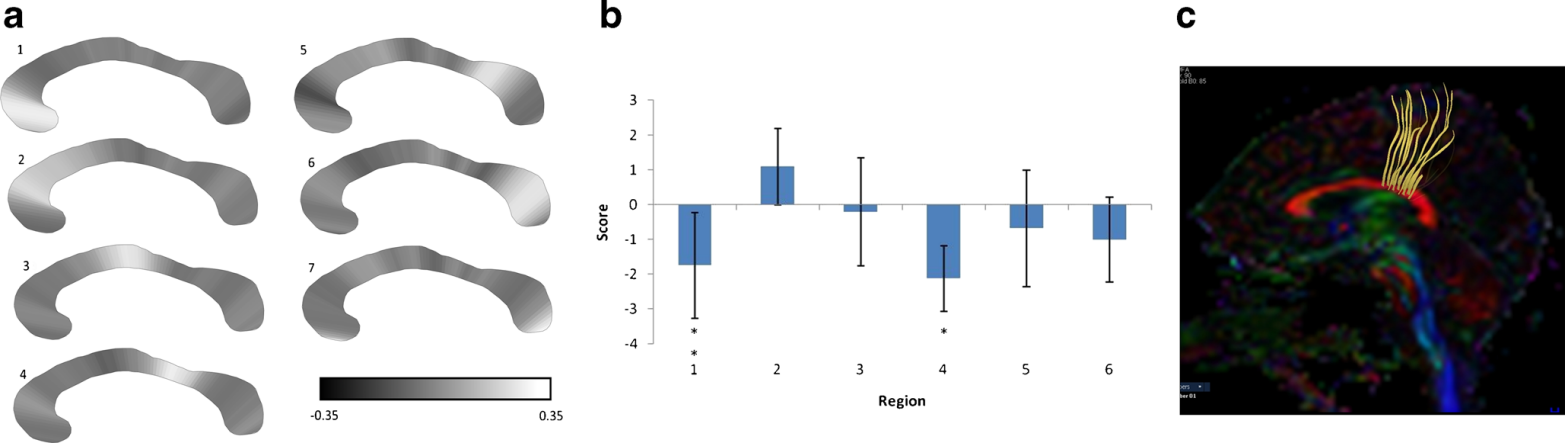
Analysis of the corpus callosum. **a** Schematic representations of the loadings of the individual component factors mapped onto a schematic corpus callosum. The grey-scale density represents the weighting of each individual width within the factor. Factors 1-7 represent, respectively, the genu, anterior body, mid body, mid-posterior body, posterior body, splenium, and the extreme anterior and posterior ends of the corpus callosum. **b** Comparison of factor scores by anatomical regions 1-6, with the factor scores interpreted as a positive value representing relative thickening of that region of the corpus callosum in children with hypoxic-ischaemic brain injury, and negative values indicating a relative narrowing. The factors corresponding to the extreme ends of the corpus callosum are not shown. Region 4, *P* < 0.01; region 1, *P* < 0.05. **c** A tractography image from a diffusion tensor imaging study in a normal adult shows that the commissural tracts implicated in the focal injury originate in the paracentral lobules

Principal components analysis of the callosal regions (Fig. 3) yielded seven factors, each with explained variance of >1% of total variance. One factor showed strong loadings for the extreme anterior and posterior aspects of the corpus callosum, which was thought to be a spurious finding related to the tapering shape (Fig. 3). The other six factors showed strong loadings to well-defined anatomical regions. Analysis of variance of the factor widths (Table 2) showed lower factor scores in the acute profound hypoxic–ischaemic brain injury group (indicative of narrowing of the corpus callosum) in region 4 (mid- to posterior body of the corpus callosum, *P* = 0.002) and in region 1 (the genu of the corpus callosum, *P* = 0.01). No other factor region demonstrated significant differences in width between the study groups.

**Table 2:** Results of analysis of variance between the patient groups using as factors anatomical regions (rostral to dorsal) of the corpus callosum, as illustrated in Fig. 3

Factors (regions of the corpus callosum)	*F*	*P-value*
Factor 1* (genu of the corpus callosum)	7.728	0.011
Factor 2	1.201	0.29
Factor 3	0.057	0.81
Factor 4* (mid to posterior body of the corpus callosum)	12.64	0.0019
Factor 5	0.7364	0.40
Factor 6	2.014	0.17

* Note that only in factors 1 (genu) and 4 (mid- to posterior body) is *F* higher than the critical value of 4.35 and hence the null hypothesis can be rejected: in these regions the difference in widths was not caused by chance variation alone

Using support vector machine classification, we found that callosal width centile analysis could be used to stratify the patients, on the basis of their imaging, into hypoxic–ischaemic brain injury group or control group with a high degree of separation (Table 3). The receiver operator characteristic curves (Fig. 3) plot the true-positive rate of classification (sensitivity) on the y-axis against the false-positive rate of classification (specificity) into either the hypoxic–ischaemic brain injury group or the control group. In both of these classifications the curves trend towards 1.

**Table 3:** Results of corpus callosum width centile support vector machine analysis between the patient groups; the results are established metrics for the assessment of machine learning experiments where tp = true positive, fp = false positive and fn = false negative

	Precision^a^	Recall^b^	F-measure^c^	AUC^d^
HIE group	1	0.909	0.952	0.955
Control group	0.917	1	0.957	0.955

^a^Precision = tp/tp + fp

^b^Recall = tp/tp + fn

^c^F-measure, a measure of classification performance, = 2 × precision × recall/precision + recall

^d^AUC (area under the curve) value refers to the receiver operating characteristic curves (Fig. 4)

*HIE* hypoxic–ischaemic encephalopathy

## Discussion

Cerebral palsy is a neurodevelopmental condition that usually becomes clinically apparent in infancy and is non-progressive throughout life [10]. The prevalence of cerebral palsy in the United States is approximately 2.4 per 1,000 live births [11] and in Europe it increased from 0.8 to 1.4 per 1,000 live births between the 1970s and the 1990s [12]. A definite cause for this increase has not been established, and it is entirely possible that this apparent increase actually reflects improved diagnosis and reporting [12]. There are many reasons a child could develop cerebral palsy and it is customary to divide the aetiological agents into prenatal, perinatal and postnatal causes [13]. Several sub-types of cerebral palsy have been determined by the clinical manifestations, and in some situations there is a relationship between the subtype and the aetiological cause or the time at which the injury occurred. For example, dyskinetic cerebral palsy most often arises as a result of perinatal injury, the two commonest causes being kernicterus and acute profound hypoxic–ischaemic brain injury. The relationship is imperfect; for example we have recently shown that approximately 50% of children with well-documented profound hypoxic–ischaemic brain injury have spastic cerebral palsy [14].

Acute profound hypoxic–ischaemic brain injury can occur secondary to severe cerebral hypoperfusion as might result from uterine rupture, for example. The resulting severe hypoxia–ischaemia tends to produce selective damage to the parts of the brain with the highest metabolic demands (probably reflecting ongoing myelination rather than neuronal activity). As such, the typical pattern of injury seen on MR imaging involves the basal ganglia and thalami and explains why dyskinetic cerebral palsy is a frequent clinical outcome. Other regions of the brain are involved, however, including the paracentral lobules, which contain the primary motor and sensory cortices and their afferent and efferent connections (Fig. 1).

In a prior publication we proposed a mechanism by which acute profound hypoxic–ischaemic brain injury could produce either dyskinetic or spastic cerebral palsy [14]. The basal ganglia injury is virtually always involved in acute profound hypoxic–ischaemic brain injury and it is likely central to the causation of dyskinetic cerebral palsy (particularly the subthalamic nucleus). However it is necessary for the primary motor cortex and its connections to be relatively well preserved in order to express the dyskinesia. If, on the contrary, the primary motor structures are severely injured, the resulting cerebral palsy is likely to be spastic in nature, the dyskinesia being masked by impairment of the final common motor pathway. This is the main reason we wished to quantify the degree of injury to the paracentral lobules. In our earlier studies we used a three-point scale based on a subjective assessment of the degree of gliosis on T2-weighted MRI in the paracentral white matter and surrounding brain. More robust, objective methods are required, and corpus callosum centile width analysis provides such a possible imaging biomarker.

The corpus callosum appears at 10–11 weeks of gestational age, first between the anterior and hippocampal commissures, and achieves a recognisable adult anatomical configuration by approximately 17 weeks. The corpus callosum is the midline crossing point for fibres arising from the cortex passing to the homotypic contralateral cortex and is vital for the integration of perceptual, cognitive, learned and volitional information [15]. Whilst measurements of the overall size of the corpus callosum are useful in some situations, focal brain pathology requires more detailed regional assessments of the corpus callosum, as is the case in the paracentral lobule involvement in acute profound hypoxic–ischaemic brain injury. Although the corpus callosum does have anatomical subregions (genu, body, splenium and rostrum) their delineation is open to subjective interpretation, so most attempts to subdivide the corpus callosum have been made on a more pragmatic basis on sagittal images [15–18]. Most studies refer to the postmortem work of Witelson, who divided the corpus callosum into specific segments [18], although this has now been revised [15] and the current corpus callosum subdivisions are based on known cortical interconnectivities (Fig. 3).

In addition to measuring the entire corpus callosum, our approach to regional measurements was based on Denenberg’s technique dividing the corpus callosum into centiles after segmentation [2, 3]. There were no significant differences in the indicators of total callosal size when the acute profound hypoxic–ischaemic brain injury group was compared with the pathology control group. Regional reduction in size of the corpus callosum was suggested on analysis of centiles but did not reach statistical significance because of wide confidence intervals [4].

**Fig. 4:**
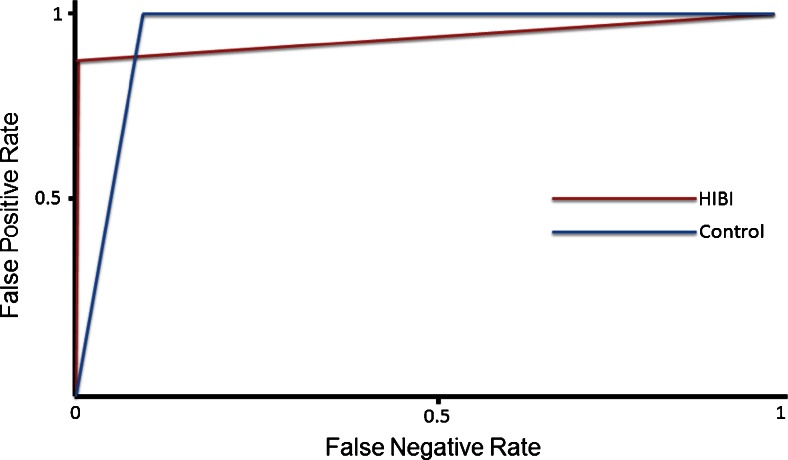
Support vector machine stratification performed on the imaging dataset for each participant with classification into one of two groups: (1) hypoxic–ischaemic brain injury (HIBI) or (2) developmental delay control. Receiver operator characteristics curves of classification (correct classification = true positive) into either the hypoxic–ischaemic brain injury group or the control group. The receiver operating characteristic curve pertaining to the stratification into each group is shown here with the associated analysis data (Table 3). Note the high degree of stratification, with an area under the curve of over 95% relating to both groups. This demonstrates the power of this technique when applied to this particular imaging metric. As such it points towards such callosal analysis in translational clinical and academic applications

Principal components analysis reduced the number of variables from 99 to 7, which could be clearly localised to distinct regions of the corpus callosum (Fig. 4). The resulting factors were inherently normalised to the overall corpus callosum size, with each factor having a sample mean of zero. The interpretation of the factors is that a positive value represents regional thickening relative to both the individual corpus callosum size and the average corpus callosum shape. Conversely, a negative value represents relative thinning of the corpus callosum. Analysis of variance of these factors demonstrated a statistically significant reduction in factor width in the acute profound hypoxic–ischaemic brain injury cases in regions 1 and 4, corresponding to the genu and mid/posterior body of the corpus callosum.

Posterior regional corpus callosum cross-sectional area reduction has been reported in pre- and perinatal injury in a pattern that mirrors that of the hemispheric lesion [19]. However there are considerable methodological differences between our study and the prior report. The prior study divided the corpus callosum into five equal-length regions, whereas our study made no a priori assumption about the regional makeup of the corpus callosum. Further, the prior study examined a heterogeneous group of children, whereas our study examined only children with acute profound perinatal hypoxic–ischaemic injury.

What is the cause of the focal changes seen in the corpus callosum of children with acute profound hypoxic–ischaemic brain injury? The corpus callosum comprises approximately 300 million axons, all of which are non-myelinated at birth. Post-natal increase in volume of the corpus callosum is caused by myelination of axons rather than new axons crossing the midline, so at least two possibilities for reduced volume exist: reduction in the number of axons because of hemispheric damage, or a reduction in the amount of myelin being deposited. Diffusion tensor imaging of children with periventricular leukomalacia (the typical abnormality associated with hypoxic–ischaemic brain injury in pre-term deliveries) has shown a statistically significant reduction in fractional anisotropy values in the genu and splenium of the corpus callosum [20]. In a similar study the reduced volume of the corpus callosum was attributed to secondary degeneration [21].

Caution must be applied when extrapolating information from cases of periventricular leukomalacia when discussing acute profound hypoxic–ischaemic brain injury in children born at term, but our working hypothesis is that the focal reduction of corpus callosum volume is caused by secondary destruction of axons rather than dysmyelination. Recent work using MR tractography studies has generally confirmed the known anatomy of the corpus callosum–neocortex interactions shown in primate studies and in some cases enabled a greater understanding of the projections to specific cortical regions in humans. Our study shows that the mid and posterior regions of the body of the corpus callosum showed specific, statistically significant reduction in volume in children with acute profound hypoxic–ischaemic brain injury and that the commissural fibres passing through that region predominantly arise from the premotor, motor and sensory cortices (Fig. 3). More specifically, visual comparison of tractographic images suggests that region 4 of the corpus callosum described in this study is the site of axon commissuration from the paracentral lobule, which includes the primary motor and sensory cortices. This finding that corpus callosum size reduction relates to the site of hemispheric white matter injury echoes the earlier conclusion of Moses et al. [19]

In our support vector machine analysis we also demonstrated the potential for this technique to be used as a potential imaging biomarker of hypoxic–ischaemic brain injury without the need for subjective operator-dependent interpretation of the callosal morphology. Our results demonstrate highly sensitive and specific automated differentiation between children with hypoxic–ischaemic brain injury and those with developmental delay (controls). This points towards rapid translational clinical application of this technique after it is validated in a prospective study alongside the collection of normal individual corpus callosum metrics. Robust classification on the basis of the callosal thinning would, of course, require future prospective validation, but these preliminary results point towards the promise of a future hypoxic–ischaemic brain injury biomarker using quantitative imaging findings obtainable from MRI imaging systems available worldwide. The adoption of quantitative biomarkers such as this is vital if we are to advance our diagnostic and investigative understanding of brain development in normal as well as pathological states.

We recognise that this study has several limitations. Most notably is the lack of a true control group, because we do not have access to MR imaging of healthy, typically developing children at this time. Our pathology control group consisted of children having clinical examinations for developmental delay but no specific focal neurological symptoms. It is acknowledged that this is not ideal, and that the use of age-matching is, in itself, potentially confounded by this. Nevertheless, such a study could result in serious confounding of the non-normalised measures if the control and study groups were not broadly representative for age. We have also not been able to distinguish between the “loss of axons” and “dysmyelination” theories of volume loss in the corpus callosum because we did not perform diffusion tensor imaging/tractography studies in these cases. Diffusion tensor imaging/tractography has been shown to be valuable in children with periventricular leukomalacia [20, 21] and warrants further study.

We cannot definitively explain the finding of volume loss in the genu of the corpus callosum. In the literature there is no evidence of imaging or functional change in the frontal lobes radiating through the genu of the corpus callosum in patients with proven hypoxic–ischaemic brain injury. However it must be noted that the assessment of these regions has been based on conventional MR imaging or pathological assessment [22]. Before we reject this finding as representing a type I statistical error because of the small sample size, it would be necessary to (1) replicate the findings herein on a larger dataset and (2) look for multi-parametric imaging correlates such as reduction in fibre density on diffusion imaging and loss of regional fractional anisotropy relating to the areas radiating through the genu of the corpus callosum. If this were found then one could rightly assume that there is loss of white matter integrity in this region but not on a scale identifiable on conventional standard MRI sequences.

This hypothesis might be borne out by the fact that when the hypoxic–ischaemic brain injury patients’ imaging was assessed for evidence of hypoxic–ischaemic brain injury by paediatric neuroradiologists blinded to the diagnosis, it was noted that in two cases there was clear evidence of basal ganglia/thalamic and posterior limb of the internal capsule T2 signal change (commensurate with the clinical and birth history diagnosis) but no paracentral T2 signal abnormality. Despite this, however, these cases were correctly characterised by the support vector machine analysis. This implies that the callosal thinning (no doubt related to tract loss in the posterior body) was not evident as conventional T2 signal abnormality relating to the fibre tracts from that region of the callosum itself. Similarly, therefore, one could extrapolate that there could be frontal lobe fibre loss that is not evident on T2-W imaging.

From a future clinical perspective, the identification and validation of quantitative biomarkers for hypoxic–ischaemic brain injury not clearly identifiable on standard MRI sequences, if correlated with features such as cellular density and fibre tracking, will become more vital as we encounter more children who have been treated for hypoxic–ischaemic brain injury through cooling and our current imaging phenotype becomes less reliable in this cohort of patients. It will be interesting to see whether we can also correlate the degree of hemispheric involvement with the degree of focal loss of volume of the corpus callosum on an individual level, but again this forms the basis for future work.

## Conclusion

The overall measurements of the corpus callosum size were not statistically significantly different between the acute profound hypoxic–ischaemic brain injury group and the pathology control group. However this is the first study to show that quantitative analysis of the corpus callosum on a regional basis demonstrates a statistically significant reduction in size of the genu and posterior body of the corpus callosum in acute profound hypoxic–ischaemic brain injury. This finding is anatomically coherent with the known selective involvement of the paracentral lobules in profound hypoxic–ischemic brain injury and forms a basis for future studies on the aetiology and significance of that injury as well as a possible biomarker for white matter volumes based on corpus callosum thickness.
